# Compatibility of intravitreally applied epidermal growth factor and amphiregulin

**DOI:** 10.1007/s10792-021-01761-w

**Published:** 2021-03-13

**Authors:** Mukharram M. Bikbov, Timur A. Khalimov, Marc Cerrada-Gimenez, Symantas Ragauskas, Giedrius Kalesnykas, Jost B. Jonas

**Affiliations:** 1 Ufa Eye Research Institute, Ufa, Bashkortostan Russia; 2 Experimentica Ltd, Kuopio, Finland; 3 Department of Ophthalmology, Medical Faculty Mannheim, Heidelberg University, Universitäts-Augenklinik, Theodor-Kutzer-Ufer 1-3, 68167 Mannheim, Germany; 4 Institute of Clinical and Scientific Ophthalmology and Acupuncture Jonas and Panda, Heidelberg, Germany

**Keywords:** Epidermal growth factor, Epidermal growth factor receptor, Amphiregulin, Toxicity, Retina, Retinal pigment epithelium

## Abstract

**Introduction:**

To examine the compatibility of intravitreally injected epidermal growth factor (EGF) and amphiregulin as EGF family member.

**Methods:**

Four rabbits (age: 4 months; body weight: 2.5 kg) received three intravitreal injections of EGF (100 ng) uniocularly in monthly intervals and underwent ocular photography, tonometry, biometry, and optical coherence tomography. After sacrificing the rabbits, the globes were histomorphometrically examined. In a second study part, eyes of 22 guinea pigs (age: 2–3 weeks) received two intravitreal administrations of amphiregulin (10 ng) or phosphate buffered solution (PBS) in 10-day interval, or were left untouched. Ten days after the second injection, the guinea pigs were sacrificed, the enucleated eyes underwent histological and immune-histological examinations.

**Results:**

The rabbit eyes with EGF injections versus the contralateral untouched eyes did not show significant differences in intraocular pressure (7.5 ± 2.4 mmHg vs. 6.8 ± 2.2 mmHg; *P* = 0.66), retinal thickness (158 ± 5 µm vs. 158 ± 3 µm; *P* = 1.0), cell counts in the retinal ganglion cell layer (3.3 ± 1.7 cells/150 µm vs. 3.0 ± 1.4 cells/150 µm; *P* = 0.83), inner nuclear layer (46.4 ± 23.2 cells/150 µm vs. 39.6 ± 6.4 cells/150 µm; *P* = 0.61), and outer nuclear layer (215 ± 108 cells/150 µm vs. 202 ± 47 cells/150 µm; *P* = 0.83), or any apoptotic retinal cells. The guinea pig eyes injected with amphiregulin versus eyes with PBS injections did not differ (*P* = 0.72) in the degree of microglial activation, and both groups did not differ from untouched eyes in number of apoptotic retinal cells and retinal gliosis.

**Conclusions:**

Intravitreal applications of EGF (100 ng) in rabbits nor intravitreal applications of amphiregulin (10 ng) in guinea pigs led to intraocular specific inflammation or any observed intraocular destructive effect. The findings support the notion of a compatibility of intraocular applied EGF and amphiregulin.

## Introduction

Epithelial growth factor (EGF) is a growth factor that stimulates cell growth, proliferation and differentiation by binding to its receptor EGFR [1]. Human EGF is a 6.4-kDa protein with 53 amino acid residues and three intramolecular disulfide bonds. EGF is member of the so called EGF family which is characterized by highly similar structural and functional characteristics and all members of which contain one or more repeats of the conserved amino acid sequence: CX7CX4-5CX10-13CXCX8GXRC (C: cysteine; G: glycine; R: arginine; X: any amino acid) [1]. Besides EGF itself, the EGF family includes the heparin-binding EGF-like growth factor (HB-EGF), the transforming growth factor-α (TGF-α), amphiregulin, epiregulin, epigen, betacellulin, and neuregulin-1, neuregulin-2, neuregulin-3, and neuregulin-4 [1]. All EGF family members contain the amino acid sequence mentioned above with three intramolecular disulfide bonds formed by the six cysteine residues. These disulfide bonds form three structural loops which are responsible for the high-affinity binding between the EGF family members and the EGFR [2].

Among numerous studies conducted to explore the characteristics and effects of EGF and the EGF family members on extraocular tissues and organs, investigations have also revealed effects of EGF and EGF family members on intraocular tissues and cells, including the retinal pigment epithelium (RPE) [3–6]. Experimental animal studies on guinea pigs and monkeys demonstrated an increase in axial elongation in the eyes of young guinea pigs with intravitreal applications of EGF family members [7–9]. Vice versa, the intravitreal application of antibodies to EGF and EGF family members and EGFR resulted in a decrease in axial elongation in young guinea pigs and young monkeys, with and without lens-induced myopization [7–9]. It has been postulated that such effects may occur via the RPE-associated enlargement of Bruch´s membrane in the equatorial regions of the eyes [10]. Other investigations on cell cultures showed a direct influence of EGF and EGF family on the proliferation and migration of RPE cells [9].

The RPE is primarily or secondarily involved in many retinal diseases, including age-related macular degeneration (AMD) and retinal dystrophies such as retinitis pigmentosa [9]. In view of the findings of the previous studies that EGF and its family members had an influence on the RPE in-vitro and in-vivo, it has been postulated that the intraocular application of EGF and its family members might theoretically have a beneficial effect on the clinical course of these retinal disorders. Since before any new clinical application of a molecule its applicability and safety have to be examined, we conducted this investigation to explore the intraocular compatibility of EGF and amphiregulin as EGF family members in a potential first step for the intraocular therapeutic application of EGF and its family members for the therapy of RPE-associated diseases.

## Methods

The first part of the study included four adult rabbits with an age of four months and a body weight of 2.5 kg. All rabbits were treated in accordance with the ARVO (The Association for Research in Vision and Ophthalmology) Statement for the Use of Animals in Ophthalmic and Vision Research. The rabbits were purchased from a commercial vendor (The Federal State Unitary Enterprise “Scientific and Production Association for Immunological Preparations “Microgen” of the Ministry of Health of the Russian Federation, Ufa Bashkortostan). They were housed at a constant temperature (22 ± 1 °C) and in a light-controlled environment (lights on from 7 am to 7 pm) with ad libitum access to food and water.

The rabbits received three intravitreal injections of 100 ng EGF into their right eyes in one-month intervals, while their left eyes remained untouched. The injections were performed in the upper right quadrant of the eyes in a distance of 3–4 mm from the limbus under general anesthesia. Anesthesia was achieved by an intramuscular injection (biceps femoris) of Zoletil® (15 mg/kg) (tiletamine mixed with zolazepam; Valdepharm Co., Val-de-Reuil, France) and xylazine (20 mg/kg) (Xyla®; Interchemie Werken, De Adelaar B.V., A Waalre, The Netherlands) and by the topical application of anesthetic eye drops (0.4% oxybuprocain, Inocain; Promed Exports, New Delhi, India). At baseline of the study, and at 2 days, 7 days, and 29 days after each injection the rabbits were examined by photography of the external eye and of the anterior and posterior segment of the eye (VISUCAM 500, Carl Zeiss Meditec AG, Jena, Germany), tonometry (Auto-2Ref/Keratometer HRK-7000A Huvitz Co, Ltd., Gyeonggi-do, Korea), and optical coherence tomography (OCT) of the posterior fundus (RetinaScan-3000, Nidek Co.,ltd., Aichi Japan).

Four weeks after the third injection, the eyes were enucleated and the rabbits were sacrificed under general anesthesia by injecting air (5–10 mL) into the ear vein. We used a mixture of 4% formaldehyde and 1% glutaraldehyde to fixate the globes immediately after they had been enucleated. After the eyes had remained in the fixative solution for 1 week at room temperature, we measured the globe dimensions in the anterior–posterior, horizontal, and vertical direction. For each globe, we removed a central part which was about 8 mm thick and included the pupil and the optic nerve head. It was dehydrated in alcohol, before we imbedded it in paraffin. We prepared slides with a thickness of 4–6 µm, and stained them with hematoxylin eosin. We counted the cell numbers in the retinal ganglion cell layer, the inner nuclear layer, and the outer retinal nuclear layer. We additionally stained the slides for apoptotic cell death using the TUNEL (terminal deoxynucleotidyl transferase dUTP nick end labeling) technique.

The second part of the study included 22 guinea pigs with an age of two to three weeks. All animals were treated in accordance with the ARVO Statement for the Use of Animals in Ophthalmic and Vision Research and the EC Directive 86/609/EEC for animal experiments, using protocols approved and monitored by the Animal Experiment Board of Finland (Experimentica Ltd. animal license number ESAVI-2017-000803). The guinea pigs (HsdDhl:Dh) were purchased from a commercial vendor (Envigo, Netherlands). We kept the animals at a constant temperature of approximately 22 °C. The environment was light-controlled with the lights on from 7 am to 7 pm. There was ad libitum access to food and water.

The animals were divided into four groups:

- Group I: Animals without lens-induced myopization and that received intravitreal injections of amphiregulin (10 ng in 5 µL) into one eye and intravitreal injections of phosphate buffered solution (PBS) into contralateral eye (*n* = 4),
- Group II: Animals without lens-induced myopization and that received intravitreal injections of amphiregulin (10 ng in 5 µL) into both eyes (*n* = 4),
- Group III: An animal without lens-induced myopization and that received intravitreal injections of amphiregulin into one eye, with the contralateral eye left untreated (*n* = 1),
- Group IV: Animals without lens-induced myopization and with both eyes left intact (*n* = 2).
- Group V: Unilateral lens-induced myopization without intravitreal injections (*n* = 6),
- Group VI: Bilateral lens-induced myopization with unilateral intravitreal injections of phosphate buffered solution (PBS) (*n *= 5).

Some of the guinea pigs underwent a lens-induced myopization for purposes other than the assessment of the compatibility of intravitreally applied amphiregulin. For achieving a lens-induced myopization, goggles with a refractive power of −10 diopters (Kang Er Ming Optical, Jiangshu province, China) were glued onto the orbital rim of both eyes. All guinea pigs with injections received two intravitreal injections of amphiregulin (R&D Systems, cat. No 989-AR) at an interval of 10 days. The intravitreal injections were performed using a 5-µL glass microsyringe (Hamilton Bonaduz AG, Bonaduz, Switzerland) under general anesthesia. The total volume of the intravitreal administration was 5 µL per eye with a dose of 10 ng/eye of amphiregulin. The injection sites were disinfected by an iodine containing solution. The injections were performed between the corneal limbus and the equator of the eyes in order to deliver the compounds to the posterior pole of the eyes. Chloramphenicol (Oftan Chlora®, Santen) ointment was applied after each injection. All these procedures were carried out under general anesthesia by an injection of a mixture containing ketamine (10 mg/kg) (Ketaminol Vet 50 mg/ml, Vnr 51 14 85, Lot A115A01; Intervet, Haar, Germany) and medetomidine (0.1 mg/kg) (Domitor 1 mg/mL, Vnr 01 56 02, Lot 1770287; Orion Pharma, Espoo, Finland). After the procedure, anesthesia was reversed by an injection of an α2-antagonist for medetomidine (0.8 mg/kg) (Antisedan 5 mg/mL, Vnr 47 19 53, Lot 1757793; Orion Pharma, Espoo, Finland). At each time point, the eyes were inspected for signs of toxicity. Ten days after the second injection, the guinea pigs were sacrificed by an intraperitoneal injection of an overdose of an anesthetics (150 mg pentobarbital/kg body weight; Mebunat Vet, Orion Pharma, Espoo Finland).

**Fig. 1:**
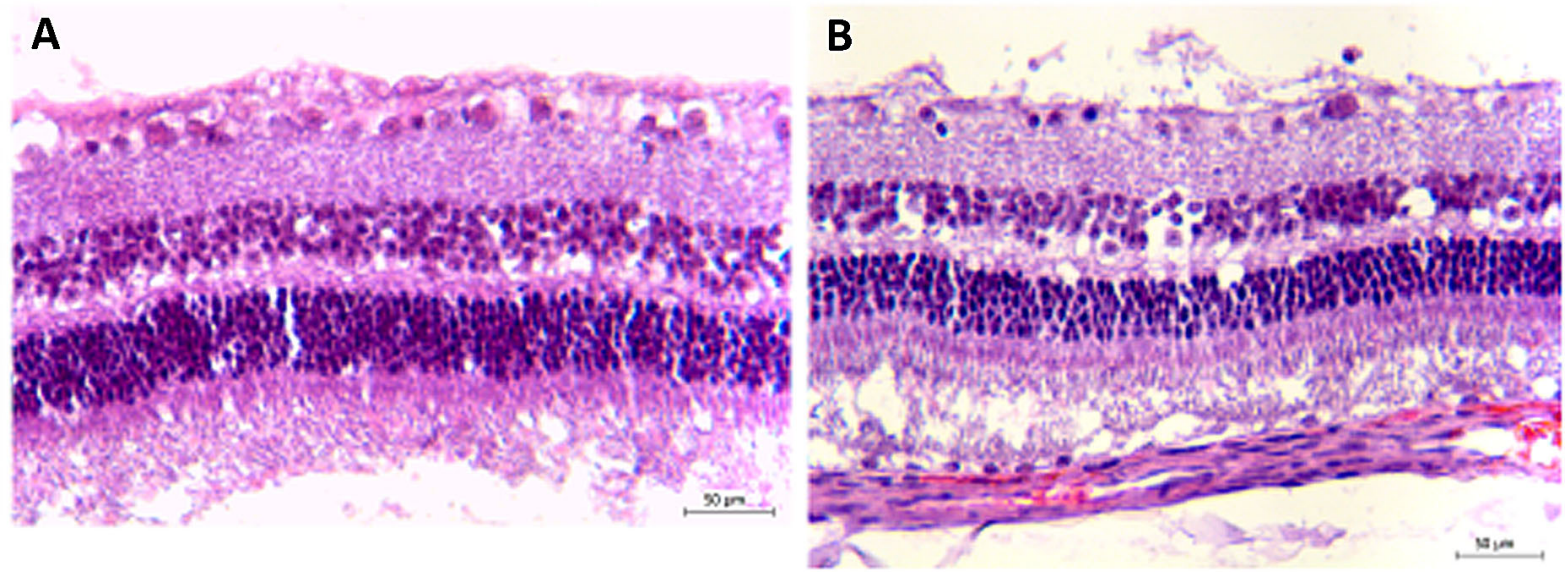
Representative examples the retinas of naive guinea pig eyes which received two intravitreal injections of phosphate buffered solution (**a**) or two intravitreal injections of amphiregulin (10 ng in 5 µL) (**b**) at an interval of 10 days, and which were sacrificed ten days after the second injection. Scale bar = 50 μm

**Fig. 2:**
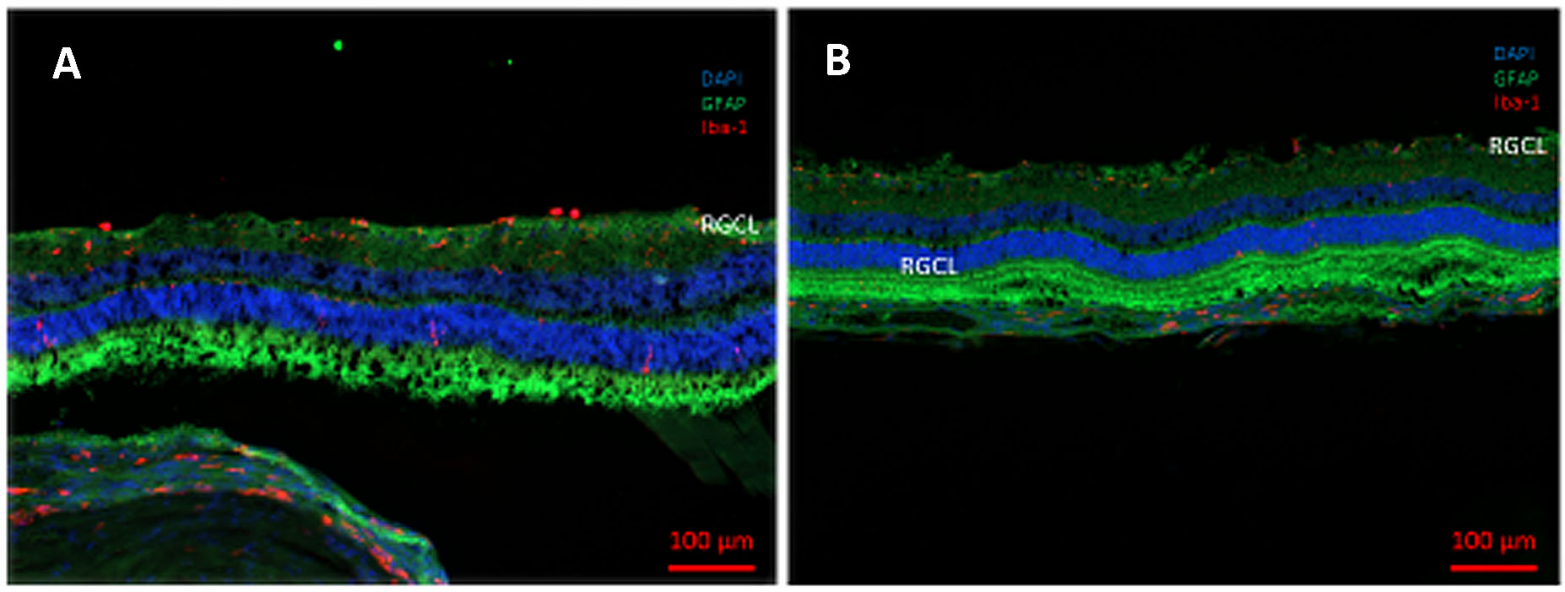
Representative examples the retinas of naive guinea pig eyes which received two intravitreal injections of phosphate buffered solution (**a**) or two intravitreal injections of amphiregulin (10 ng in 5 µL) (**b**) at an interval of 10 days, and which were sacrificed ten days after the second injection. Micrographs from sections immunostained against microglial marker Iba-1 (ionized calcium-binding adapter molecule 1) (in red) and glial fibrillary acidic protein (GFAP, in green). Nuclei were labeled with DAPI (4′,6-Diamidin-2-phenylindol) (blue). Scale bar is 100 µm

**Fig. 3:**
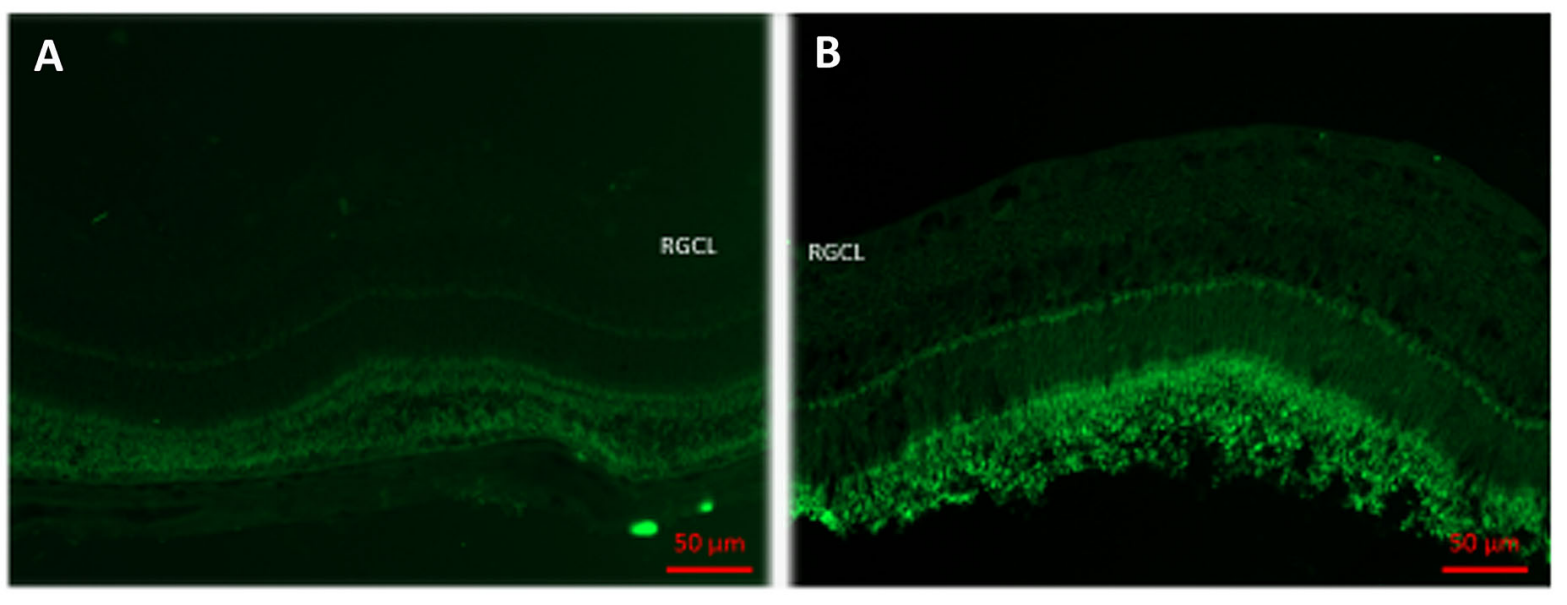
Representative examples the retinas of naive guinea pig eyes which received two intravitreal injections of phosphate buffered solution (**a**) or two intravitreal injections of amphiregulin (10 ng in 5 µL) (**b**) at an interval of 10 days, and which were sacrificed ten days after the second injection. Staining by TUNEL. *Note* No increase in TUNEL-positive cells (in green) was detected in the retinal ganglion cell layer, retinal inner nuclear layer, and retinal outer nuclear layer. *RGCL* retinal ganglion cell layer; Scale bar is 50 µm

Ocular biometry (Aviso, The Ultrasound Platform; Quantel Medical, 63808 Cournon d'Auvergne, France) was carried out under general anesthesia at baseline, prior to each injection, and prior to the sacrifice of the guinea pigs. Immediately after the sacrifice of the guinea pigs, the eyes were enucleated, post-fixed overnight in 4% paraformaldehyde, and embedded in optimal cutting temperature compound, cryosectioned and immunostained against glial activation marker (glial fibrillary acidic protein (GFAP); Sigma Aldrich Co., cat no. G3893; St. Louis, MO, USA), microglial cell marker (ionized calcium-binding adapter molecule 1 (IBA1); Wako Chemicals Inc., cat. no. 019–19741; Richmond, VA, USA), native guinea pig amphiregulin (Bioss Antibodies, cat. no. BS-3847R; Woburn, MA, USA), and injected amphiregulin (R&D Systems, AF989; Minneapolis, MN, USA); or they were embedded in paraffin, sectioned and stained with hematoxylin and eosin for general histologic examination and histomorphometric retinal thickness measurements (Figs. 1, 2). In addition, the tissue was stained for apoptotic cell death detection applying the TUNEL (terminal deoxynucleotidyl transferase dUTP nick end labeling) method (Hoffmann-La Roche, cat. no. 11684795910; Basle, Switzerland) (Fig. 3).

The statistical analysis was performed using a commercially available software program (SPSS for Windows, version 25.0, IBM-SPSS, Chicago, IL). We calculated the mean and standard deviations of the main outcome parameters. We then tested the statistical significance of differences between both eyes of the same animals using the Student's t test for paired samples. A *P* value < 0.05 was considered to indicate statistical significance.

## Results

The rabbit eyes injected with EGF and the contralateral untouched rabbit eyes did not differ significantly in intraocular pressure (baseline: 6.8 ± 2.5 mmHg vs. 5.8 ± 1.0 mmHg; *P* = 0.50; end of follow up: 7.5 ± 2.4 mmHg vs. 6.8 ± 2.2 mmHg; *P* = 0.66), and retinal thickness (152 ± 5 µm vs. 157 ± 3 µm; *P* = 0.15; end of follow up: 158 ± 5 µm vs. 158 ± 3 µm; *P* = 1.0) (Table 1). In a similar manner, both groups did not vary significantly (all *P* > 0.05) in the cell count in the retinal ganglion cell layer, retinal inner nuclear layer and retinal outer nuclear layer as measured at the posterior pole, the midpoint between the posterior pole and the equator, the equator, and close to the ora serrata. They neither differed in the cross-section area of the ciliary body as a whole and of the ciliary muscle itself (Table 2). In the TUNEL staining, apoptotic cells were detected neither in the right eyes nor in the left eyes.

**Table 1:** Intraocular pressure and retinal thickness in rabbits receiving three intravitreal injections of 100 ng epidermal growth factor (EGF) into their right eyes in one-monthly intervals, while their left eyes remained untouched

Animals		Baseline examination and first injection	First follow-up examination and second injection	Second follow-up examination and third injection	Third follow-up examination and sacrifice
*Retinal thickness at the posterior pole (µm)*					
1	Right eye/left eye	152/161	156/161	160/163	153/158
2	Right eye/left eye	149/155	145/158	149/161	155/161
3	Right eye/left eye	147/158	150/163	155/159	159/158
4	Right eye/left eye	159/154	157/159	162/155	164/154
*Intraocular pressure (mm Hg)*					
1	Right eye/left eye	7/5	6/5	8/4	11/5
2	Right eye/left eye	4/6	6/10	4/3	7/6
3	Right eye/left eye	10/7	12/10	12/9	6/10
4	Right eye/left eye	6/5	3/7	2/5	6/6

**Table 2:** Cell counts in the various retinal layers and cross-section area of the ciliary body in rabbits, receiving three intravitreal injections of 100 ng epidermal growth factor (EGF) into their right eyes in one-monthly intervals, while their left eyes remained untouched

Parameters	Right eyes	Left eyes	*P* value
Cell density retinal ganglion cell layer (cells/per ocular field of view area); posterior pole	3.3 ± 1.7	3.0 ± 1.4	0.83
Cell density retinal ganglion cell layer (cells/per ocular field of view area); midpoint posterior pole to equator	3.5 ± 1.1	1.9 ± 0.9	0.06
Cell density retinal ganglion cell layer (cells/per ocular field of view area); equator	1.8 ± 1.9	3.1 ± 2.3	0.40
Cell density retinal ganglion cell layer (cells/per ocular field of view area); close to ora serrata	3.3 ± 2.1	0.8 ± 0.3	0.17
Cell density inner nuclear layer (cells/per ocular field of view area); posterior pole	38.3 ± 23.3	40.8 ± 15.6	0.89
Cell density inner nuclear layer (cells/per ocular field of view area); midpoint posterior pole to equator	46.4 ± 23.2	39.6 ± 6.4	0.61
Cell density inner nuclear layer (cells/per ocular field of view area); equator	36.1 ± 10.8	28.8 ± 10.4	0.36
Cell density inner nuclear layer (cells/per ocular field of view area); close to ora serrate	19.0 ± 5.0	19.3 ± 6.2	0.95
Cell density outer nuclear layer (cells/per ocular field of view area); posterior pole	215 ± 108	202 ± 47	0.83
Cell density outer nuclear layer (cells/per ocular field of view area); midpoint posterior pole to equator	165 ± 55	206 ± 17	0.23
Cell density outer nuclear layer (cells/per ocular field of view area); equator	161 ± 53	168 ± 28	0.84
Cell density outer nuclear layer (cells/per ocular field of view area); close to ora serrate	74 ± 3	85 ± 17	0.38
Ciliary body, total cross-section area (mm^2^)	43.9 ± 16.3	33.8 ± 13.7	0.38
Ciliary body, ciliary muscle cross-section area (mm^2^)	16.6 ± 11.0	12.3 ± 7.9	0.55

The intravital examinations of the external eye and the inner parts of the globes did not reveal any sign of intraocular inflammation, toxicity or development of cataract, such as cells or a Tyndall phenomenon in the anterior chamber aqueous, a hazy insight into the eye and onto the retina, or retinal infiltrates.

In the second part of the study, the guinea pigs from the different groups did not differ significantly (*P* > 0.20) in their weight at any time point analyzed. No significant (all *P* > 0.50) increase in TUNEL positive cell numbers and retinal gliosis was observed in the eyes injected with amphiregulin as compared to the eyes injected with PBS or the untouched eyes (Table 3). The guinea pig eyes injected with amphiregulin versus the eyes with PBS injections did not differ (*P* = 0.72) in the degree of microglial activation, while both groups (amphiregulin group, *P* = 0.004; PBS-group, *P* = 0.01) showed a significantly higher degree of microglial activation as the eyes without intravitreal injections. There was no detected difference between guinea pigs with lens-induced myopization and guinea pigs without lens-induced myopization in the number of TUNEL positive cells and in retinal gliosis (all *P* > 0.50), and in microglial infiltration (*P* = 0.35).

**Table 3:** Results of the histomorphometric examinations of guinea pig eyes undergoing intravitreal injections of amphiregulin, amphiregulin antibody, or phosphate buffered solution (PBS)

Animal ID	Eye	Myopia induction	Treatment	Hematoxylin and eosin staining	TUNEL staining	GFAP staining	Iba-1 staining
*Group I*							
M25	OD	Naive	Amphiregulin	Normal	−	−	+
M25	OS	Naive	PBS	Normal	−	−	+ +
M29	OD	Naive	Amphiregulin	Normal	−	−	+ + +
M29	OS	Naive	PBS	Normal	−	−	+ + +
M30	OD	Naive	Amphiregulin	Normal	−	−	+ +
M30	OS	Naive	PBS	Normal	−	−	+
M31	OD	Naive	Amphiregulin	Normal	−	−	+
M31	OS	Naive	PBS	Normal	−	−	+
*Group II*							
M8	OD	Naive	Amphiregulin	Normal	−	−	+
M8	OS	Naive	Amphiregulin	Normal	−	−	+ +
M10	OD	Naive	Amphiregulin	Normal	−	−	+ + +
M10	OS	Naive	Amphiregulin	Normal	−	−	+ +
M14	OD	Naive	Amphiregulin	Normal	−	−	+ +
M14	OS	Naive	Amphiregulin	Normal	−	−	+ +
M16	OD	Naive	Amphiregulin	Normal	−	−	+
M16	OS	Naive	Amphiregulin	Normal	−	−	+
*Group III*							
M36	OD	Naive	Amphiregulin	Normal	−	−	+ +
M36	OS	Naive	Naive	Normal	−	−	+
*Group IV*							
M12	OD	Naive	Naive	Normal	−	−	+
M12	OS	Naive	Naive	Normal	−	−	+ +
M15	OD	Naive	Naive	Normal	−	−	+
M15	OS	Naive	Naive	Normal	−	−	+
*Group V*							
M26	OD	Unilateral (OD)	Untreated	Normal	−	−	+
M26	OS	No	Untreated	Normal	−	–	+
M27	OD	Unilateral (OD)	Untreated	Normal	−	−	+
M27	OS	No	Untreated	Normal	−	−	+
M28	OD	Unilateral (OD)	Untreated	Normal	−	−	+
M28	OS	No	Untreated	Normal	−	−	+
M32	OD	Unilateral (OD)	Untreated	Normal	−	−	+
M32	OS	No	Untreated	Normal	−	−	+
M33	OD	Unilateral (OD)	Untreated	Normal	−	−	+
M33	OS	No	Untreated	Normal	−	−	+
M34	OD	Unilateral (OD)	Untreated	Normal	−	−	+
M34	OS	No	Untreated	Normal	−	−	+
*Group VI*							
M9	OD	Bilateral	PBS	Normal	−	−	+ +
M9	OS	Bilateral	Naive	Normal	−	−	+
M11	OD	Bilateral	PBS	Normal	−	−	+ +
M11	OS	Bilateral	Naive	Normal	−	−	+
M17	OD	Bilateral	PBS	Normal	−	−	+
M17	OS	Bilateral	Naive	Normal	−	−	+
M19	OD	Bilateral	PBS	Normal	−	−	+ + +
M19	OS	Bilateral	Naive	Normal	−	−	+
M21	OD	Bilateral	PBS	Normal	−	−	+ +
M21	OS	Bilateral	Naive	Normal	−	−	+

TUNEL (terminal deoxynucleotidyl transferase dUTP nick end labeling) grading: −, no cells or max. 1 TUNEL positive cell per 100 µm retinal length;  + , 2–5 TUNEL positive cells;  +  + , 6–10 TUNEL positive cells;  +  +  + , > 10 TUNEL positive cells

GFAP (glial fibrillary acidic protein) grading: −GFAP is localized exclusive in the nerve fiber layer (NFL);  + , GFAP is present also in Muller cells in addition to the NFL immunoreactivity

Iba-1 (ionized calcium-binding adapter molecule 1) grading:  + , 1–2 Iba-1 positive cells per 100 um of retinal length; +  + , 3–5 Iba-1 positive cells; +  +  + , > 5 Iba-1 positive cells

## Discussion

Neither in the rabbits nor in the guinea pigs, any injection-related effects such as a loss of retinal cells, a change in the intravitally measured OCT-based retinal thickness measurements, an increase in the number of apoptotic retinal cells, a shrinkage or swelling of the ciliary body, and induction of astrogliosis, or changes in intraocular pressure were noted. In the same manner, there were no signs of intravitreal inflammation detected, neither during the intravital examinations nor upon histological examinations of the globes. The degree of the microglial activity as measured by the positivity for IBA-1 does not differ significantly (*P* = 0.72) between the guinea eyes with amphiregulin injections and the guinea pigs with PBS injections, so that the increased microglial activity in the group of eyes with intravitreal injections as compared to the group of eyes with intravitreal applications might have been due to the procedure of the intravitreal injection. The findings support the notion, that repeatedly intravitreally applied EGF and amphiregulin did not result in a specific intraocular inflammatory or toxic effect. It supports the notion of an intraocular compatibility of intravitreally applied EGF and amphiregulin.

Previous in-vitro studies on ocular effects of EGF and amphiregulin in human RPE cells revealed that EGF induced the EGF-EGFR-MAPK (mitogen-activated protein kinase) signal transduction pathway in a concentration-dependent manner. It suggests that EGF is involved in the activation of the proliferation and migration of human RPE cells [4]. Other studies showed that RPE cell-derived HB-EGF stimulated the retinal wound healing [3]. It was reported that the survival of RPE D407 cells as induced by EGF was associated with a signaling through the PI3K (phosphatidylinositol 3′-kinase) pathway and the ERK/MAPK (MEK-dependent mitogen-activated kinase) pathway [11]. In a study by Zhang and colleagues, EGF increased in a dose dependent manner the in-vitro wound healing and migration of human RPE cells (cell line ARPE19). The wound healing and migration of the RPE cells were reduced by a pre-treatment with inhibitors of EGFR, PI3K or AKT [12]. It was concluded that the ARPE-19 cell migration was mediated by EGF through the EGFR/PI3K/AKT signaling pathway [12]. In other studies, proliferation and migration of cultured human RPE cells were increased by EGF in a concentration-dependent manner [4, 9]. The expression of EGFR protein and mRNA in RPE cells was also increased by EGF. Using immunoprecipitation and Western blotting, a cell culture study with exposure of RPE cells to EGF in a concentration of 50 ng/ml showed an enhancement of the RPE cell survival by nearly 40% parallel to an increase in the tyrosine phosphate content of the EGFR [11]. Adding the EGFR-selective tyrosine kinase inhibitor AG1478 to the cell culture resulted in a blockage of both effects. The study also demonstrated that EGF increased the phosphorylation of the PI3K-dependent effector kinase Akt and of MAPK. As a corollary, adding the PI3K inhibitor LY294002 or the MEK inhibitor U0126 to the cell culture resulted in a reduction of the EGF-induced protection of the RPE cells. These observations led to the conclusion that an EGF-stimulated survival of RPE cells was due to a signaling through both the PI3K and ERK/MAPK pathways [11]. Other studies showed that EGF stimulated the RPE cell proliferation, an effect which was annihilated by isoproterenol or dibutyryl C-AMP [13]. Interestingly, dibutyryl C-AMP by itself decreased the RPE cell proliferation, while, in contrast, isoproterenol by itself did not have any effect on the RPE cell proliferation [13]. It was concluded that after stimulation of EGF receptors, tyrosine-kinase-activated products might have an effect on secondary messenger molecules produced due to activation of beta 2-type (linked with C-AMP formation) and muscarinic (linked with InsPs production) receptors in RPE cells [13].

These investigations have been corroborated by observations made in experimental studies on guinea pigs and young monkeys. If young guinea pigs received unilateral intravitreal injections of amphiregulin or other EGF family members, the axial elongation was more marked in the eyes injected with amphiregulin or the EGF family members as compared to the contralateral eyes which received intravitreal applications of PBS. As a corollary, eyes with intraocular applications of antibodies to amphiregulin or other EGF family members showed a decrease in axial elongation as compared to the contralateral eyes with PBS injections [7–9]. In a similar manner, young monkeys which received intravitreal injections of amphiregulin antibody showed a reduction in axial elongation in the injected eyes (own observations). These observations suggested that EGF and its family members are involved in the process of emmetropization and myopization. It may open the theoretical possibility to use intravitreally applied EGF and its family members in situations in which a marked axial hyperopia may otherwise develop or persist. In previous studies, various doses of amphiregulin have been used in animal studies. These doses ranged from 0.25 ng over 0.50 ng to 1.0 ng in the study by Jiang and colleagues, and it ranged between 1 ng over 10 ng and 20 ng in the study by Dong and associates.^7–8^.

The dose of 10 ng amphiregulin used in our study on the guinea pigs is in the upper range of the doses of amphiregulin applied in the previous investigations.

Parallel to the presumed influence of EGF on axial elongation, the EGF family members may play a role in the physiology and pathophysiology of the RPE as suggested by the cell culture studies and molecular biological examinations mentioned above. Since the RPE is primarily or secondarily involved in the course of several retinal diseases such as AMD and retinitis pigmentosa, the intravitreally applied EGF and its family members may theoretically have an influence on the course of such diseases. Interestingly, an increased prevalence of AMD was associated with axial hyperopia in several population-based studies such as the Beijing Eye Study, the Singapore Malay Eye Study, and the Central India Eye and Medical Study [14–18]. The findings may weakly suggest a co-incidence of axial hyperopia and AMD in association with a relative lack of EGF. If this assumption is valid, it may promote the potential use of EGF in the prevention or therapy of EGF. Since animal models of non-exudative AMD have been scarce and since any clinical application of a new drug necessitates examinations of its safety, the present investigation may be a first step in the evaluation of EGF and its family members for a potential intraocular use. The study showed that upon histomorphometry, immunohistochemistry and intravital examinations, inflammatory or toxic effects in associations with and specific for EGF and amphiregulin when applied intravitreally were not detected. Although the present study will not suffice for a conclusion on the safety of intravitreally applied EGF and its family members, it may give an initiative to further explore the suitability of intraocularly applied EGF and its family members for the therapy of RPE-associated disorders.

In conclusion, intravitreal applications of EGF (100 ng) in rabbits nor intravitreal applications of amphiregulin (10 ng) in guinea pigs led to intraocular specific inflammation or any observed intraocular destructive effect. The findings support the notion of a compatibility of intraocular applied EGF and amphiregulin.

## Data Availability

The datasets used and/or analyzed during the current study are available from the corresponding author on reasonable request.
